# The Role of Backbone Hydrogen Bonds in the Transition State for Protein Folding of a PDZ Domain

**DOI:** 10.1371/journal.pone.0095619

**Published:** 2014-04-18

**Authors:** Søren W. Pedersen, Greta Hultqvist, Kristian Strømgaard, Per Jemth

**Affiliations:** 1 Department of Medical Biochemistry and Microbiology, Uppsala University, Biomedical Center, Uppsala, Sweden; 2 Department of Drug Design and Pharmacology, University of Copenhagen, Copenhagen, Denmark; University of South Florida College of Medicine, United States of America

## Abstract

Backbone hydrogen bonds are important for the structure and stability of proteins. However, since conventional site-directed mutagenesis cannot be applied to perturb the backbone, the contribution of these hydrogen bonds in protein folding and stability has been assessed only for a very limited set of small proteins. We have here investigated effects of five amide-to-ester mutations in the backbone of a PDZ domain, a 90-residue globular protein domain, to probe the influence of hydrogen bonds in a β-sheet for folding and stability. The amide-to-ester mutation removes NH-mediated hydrogen bonds and destabilizes hydrogen bonds formed by the carbonyl oxygen. The overall stability of the PDZ domain generally decreased for all amide-to-ester mutants due to an increase in the unfolding rate constant. For this particular region of the PDZ domain, it is therefore clear that native hydrogen bonds are formed after crossing of the rate-limiting barrier for folding. Moreover, three of the five amide-to-ester mutants displayed an increase in the folding rate constant suggesting that the hydrogen bonds are involved in non-native interactions in the transition state for folding.

## Introduction

Protein domains usually fold in a highly co-operative manner with concomitant formation of hundreds of non-covalent bonds. The transition state for a typical protein folding reaction looks like a distorted version of the native state as determined from experimental Φ value analyses and molecular dynamics simulations [1]. Most experimental analyses of folding transition states use the strategy of truncating side-chains of amino acid residues and probing the effect on protein folding kinetics as well as the stability at equilibrium (Φ value analysis) [2]. This approach provides information on the relative energetics of the interactions of the side-chains in the transition and ground states. However, hydrogen bonds from backbone amides also play important roles in the folding and stability of proteins although their contribution is difficult to evaluate and their contribution is currently debated [3]. However, by introducing amide-to-ester or amide-to-thioether mutations in the backbone of the protein, it is possible to assess the contribution of these hydrogen bonds to stability [4] and folding kinetics [5], [6]. Only very few proteins have been subjected to this approach, for example WW domain variants, for which the role of hydrogen bonds for overall stability as well as folding mechanism was assessed [5], [6].

**Figure 1:**
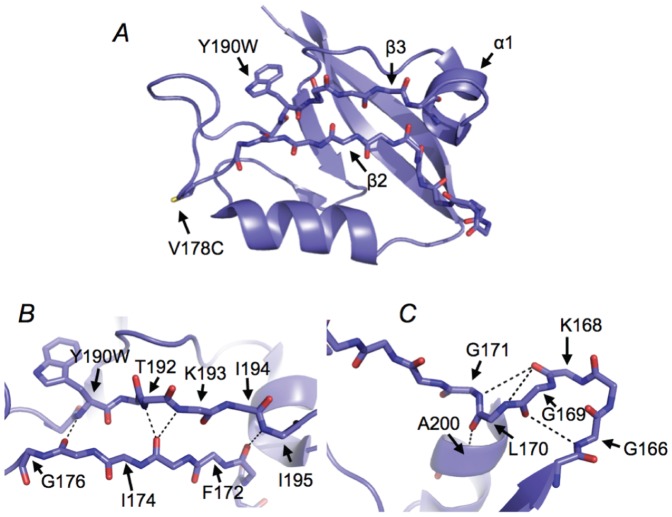
Structural basis for the amide-to-ester mutations. (*A*) The X-ray crystal structure of PSD-95 PDZ2 from the PDZ1-2 tandem [26], with the two engineered residues highlighted; Trp190 was used as a fluorescent probe and Cys178 for the semisynthesis of backbone amide-to-ester containing mutants. (*B*) and (*C*) close-ups showing the backbone hydrogen bonds perturbed by the amide-to-ester mutations (dashed lines).

The PSD-95/Discs large/ZO-1 (PDZ) domains make up a family of globular adaptor protein domains, involved in signalling and scaffolding [7]. PDZ domains bind peptide segments of target proteins, often at the C-terminus [8]–[10]. This protein family has also served as a model system for detailed studies of protein folding [11]–[15]. These studies include detailed models of the transition states of folding characterized by conventional Φ-value analyses [16]–[18]. In the present paper, we use amide-to-ester mutations to investigate the formation of hydrogen bonds in the transition state for folding of the β-sheet formed by β2 and β3 strands in the second PDZ domain of PSD-95 (PSD-95 PDZ2) (Fig. 1).

This β-sheet is involved in the physiological function of PDZ domains by binding to disordered C-termini of protein ligands though backbone-backbone interactions thus forming an extended β-sheet [19]. We also probed putative backbone hydrogen bonds in the adjacent important carboxylate-binding loop, which is fully preserved among PDZ domains and essential for coordinating the C-terminal carboxylate of the protein ligands. Our data show that backbone hydrogen bonds contribute to the overall stability and form late in the folding reaction. Interestingly, some backbone hydrogen bonds appear to form non-native interactions in the transition state that slow down the folding reaction, suggesting that a certain degree of frustration [20] is present for formation of this β-sheet, which form part of the binding pocket for PDZ domains.

## Methods

### Protein constructs

The PSD-95 PDZ2 construct was similar to the one used in earlier folding studies [13], [15], containing residues 155–249 of human PSD-95 including a mutation, Y190W in β3 to probe folding by fluorescence spectroscopy. To make amide-to-ester substitutions in the backbone, an additional mutation, (V178C) was introduced. Thus, the pseudo wild type in the folding experiments in this paper is the double mutant of PSD-95 PDZ2, V178C/Y190W. Semisynthesis of the PSD-95 PDZ2 V178C/Y190W amide-to-ester mutants was performed as previously described [21].

### Equilibrium and kinetic experiments

All experiments were performed at 25°C in 50 mM potassium phosphate, pH 7.0, and 0.4 M sodium sulphate. Equilibrium denaturation of each of the PSD-95 PDZ2 mutants was performed by monitoring the change in fluorescence at 340 nm (excitation wavelength = 280 nm) at increasing concentration of urea. The data displayed the typical sigmoidal shape of an apparent two-state transition and was fitted to the appropriate equation for solvent denaturation [22]. The most destabilized mutants did not give a well-defined native baseline in the experiments but the mid-point of the transition was well defined and fitting of the data to a constrained *m*_D-N_ value was used to obtain Δ*G*_D-N_.

Kinetic experiments were performed in a stopped flow instrument (Applied Photophysics SX-17, upgraded to SX-20, Leatherhead, UK). For unfolding experiments, protein-buffer solutions were mixed with urea-buffer at different final concentrations of urea. For refolding experiments, protein-buffer-urea solutions were mixed with buffer-urea. Excitation was at 280 nm and mono-phasic kinetic traces were monitored using a 320 nm cut-off filter. Observed rate constants for (un)folding were measured over a wide range of urea concentration (0–8 M) and analysed as described in the following section.

### Analysis of kinetic data

The folding data of the PDZ variants were analysed using an equation, which takes into account a change of rate-limiting step manifested as a kink in the refolding arm of the chevron plot:

log *k*_obs_ = log (*k*_U2_ exp (*m*_TOT_(1- *β*_T2_)[U]/*RT*)+*k*_F1_ exp (-(*β*_T1_ *m*_TOT_)[U]/*RT*)/(1+*k*_F1_/*k*_F2_ exp (*m*_TOT_ (*β*_T2_-*β*_T1_)[U]/*RT*))), where *RT* is 0.59 kcal mol^−1^ at 25°C and [U] is the concentration of urea. The total kinetic *m*-value *m*_TOT_ is related to the sum of the slopes of log *k*_obs_ versus [U] [23]. For example, in a system where we assume that the denatured state is always in fast equilibrium with the folded state, without accumulation of intermediates (two-state), *m*_TOT_ is the sum of the slopes of log *k*_F2_ and log *k*_U2_ versus urea concentration. See Hultqvist et al. [15] and Calosci et al. [16] for details on the equation used in the kinetic analysis.

Two Φ values could be calculated for the present data set, one for the very early first transition state, TS1: Φ_TS1_ = ΔΔ*G*_D-TS1_/ΔΔ*G*_D-N_ = *RT* ln (*k*_F1_^WT^/*k*_F1_^mut^)/ΔΔ*G*_D-N_, and one for the second transition state, TS2: Φ_TS2_ = ΔΔ*G*_D-T2_/ΔΔ*G*_D-N_ = 1-ΔΔ*G*_T2-N_/ΔΔ*G*_D-N_ = 1- *RT* ln (*k*_U2_^mut^/*k*_U2_^WT^)/ΔΔ*G*_D-N_. ΔΔ*G*_D-N_ is the change upon mutation in free energy between the denatured state and the native state, and was obtained from equilibrium denaturation experiments with a constrained *m*_D-N_ value. Analysis of kinetic data was performed using Prism (GraphPad Software, Inc.) and Kaleidagraph (Synergy Software).

## Results

We designed and synthesized five different amide-to-ester mutants of PSD-95 PDZ2, L170λ, G171γ, F172φ, I174ι and G176γ as described [21] (see Powers et al. [24] for nomenclature of amide-to-ester mutations). In general, such backbone mutations remove a hydrogen bond formed by the NH of the amide and destabilize the hydrogen bond formed by the amide carbonyl oxygen [3], [24]. In the case of PSD-95 PDZ2 [25], [26], the amide NH of Phe172, Ile174 and Gly176 are posed for binding a peptide ligand and not directly involved in intra-domain hydrogen bonds. However, the carbonyl oxygen of these peptide bonds are directly involved in backbone hydrogen bonds with the neighbouring strand, β3 (Fig. 1B). Likewise, the carbonyl oxygen of the Leu170-Gly171 peptide bond forms a hydrogen bond to the backbone of Ala200, which pins the α1 helix to the carboxylate-binding loop (Fig. 1C). The carbonyl oxygen of the Gly169-Leu170 peptide bond appears to form a hydrogen bond to the amide of Gly166, thereby stabilizing the carboxylate-binding loop. Finally, the amide NH of Leu170 and Gly171 might be involved in hydrogen bonding with the backbone carbonyl of Lys168, which would also contribute to the stability of the carboxylate-binding loop (Fig. 1C). Semisynthesis of the PSD-95 PDZ2 amide-to-ester mutants required a Cys residue (Cys178) C-terminal of the backbone mutations [21]. The five amide-to-ester mutants as well as Y190W and the V178C/Y190W double mutant were all subjected to equilibrium and kinetic folding experiments. In our experiments, the V178C/Y190W mutant is considered the pseudo wild type to which the other mutants are compared.

### Circular dichroism and equilibrium denaturations

**Figure 2:**
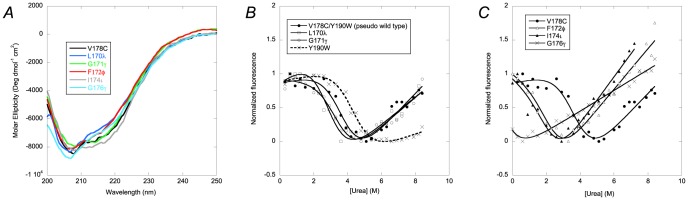
Stability of wild type and amide-to-ester variants. (*A*) Far-UV circular dichroism spectra and (*B–C*) urea-induced denaturation of wild type and amide-to-ester mutants of PSD-95 PDZ2. See Table 1 for ΔΔ*G*_D-N_ values from the curve fitting in panel B.

The V178C as well as all the amide-to-ester mutations destabilized the protein. To enable a comparison of all mutants and wild type PDZ domain, we performed folding experiments in the presence of 0.4 M sodium sulphate, which stabilized the amide-to-ester mutants such that all were folded in the absence of denaturant, as shown by urea denaturation and far-UV circular dichroism (CD) experiments (Fig. 2). However, three mutants, L170λ, F172φ and G176γ displayed a CD spectrum with a slightly lower signal at 220 nm. Nevertheless, all mutants bound a peptide ligand in a fluorescence polarization experiment [21], showing that they populate a functional PDZ conformation. The amide-to-ester mutations destabilized the protein by 0.5–2 kcal mol^−1^, with F172φ and I174ι being the most destabilizing mutations (Fig. 2). The fluorescence of Trp190 was strongly affected by the G176γ mutation, which precluded a quantitative analysis of the equilibrium denaturation experiment of this amide-to-ester mutant. The likely reason for the severe effect on the fluorescence of the G176γ mutation is that the Ala175 carbonyl oxygen binds to the backbone amide of Trp190 and its removal changes the local structure and perturbs the fluorescence of Trp190.

### Kinetic folding experiments

**Figure 3:**
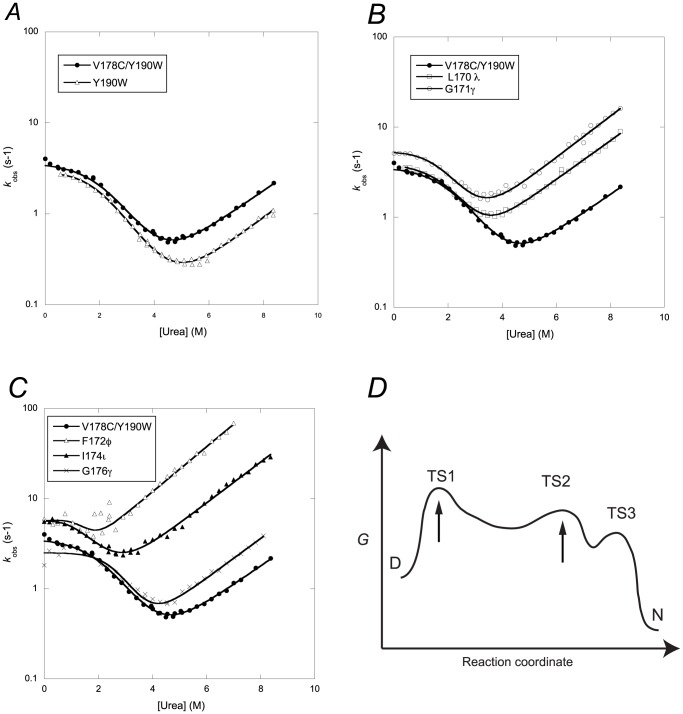
Kinetic folding experiments for wild type and amide-to-ester variants. (*A–C*) The dependence of the observed rate constant for (un)folding, *k*_obs_, for V178C/Y190W and the amide-to-ester mutants. (*D*) Free energy diagram of the folding landscape of PDZ domains at low urea concentration at which TS1 is rate limiting for the folding reaction. The arrows indicate the two transition states that were probed by the amide-to-ester mutations. See Hultqvist et al. [15] for a detailed analysis of the folding kinetics and four-state model.

The pseudo wild type and mutants of PSD-95 PDZ2 were rapidly mixed with urea-buffer solutions using a stopped-flow instrument. The resulting kinetic traces were followed by monitoring the change in Trp fluorescence and were fitted to a single exponential equation to obtain the observed rate constants (*k*_obs_) for unfolding or refolding. The *k*_obs_ values were plotted versus urea concentration to obtain so-called chevron plots (Fig. 3).

Chevron plots of two-state folders i.e., proteins that populate only two states: the denatured and native states, separated by an energetic barrier, appears as perfectly V-shaped, due to the linear dependence of the logarithm of (un)folding rate constants with denaturant concentration [27]. The chevron plots for both the pseudo wild type and the amide-to-ester mutants displayed a curvature in the refolding arm in agreement with previous folding experiments on PDZ domains [13], [15]. This curvature was interpreted as a change in rate-limiting step between two transition states, which are separated by a high-energy intermediate. The intermediate may transiently accumulate, but this cannot be distinguished in a kinetic analysis since different mechanistic scenarios give very similar mathematical solutions [27]. Therefore, only two microscopic rate constants can be appropriately determined from the present dataset, namely, the refolding rate constant *k*_F1_ (reflecting the crossing of the first barrier) and the unfolding rate constant *k*_U2_ (reflecting the crossing of the second barrier). The refolding rate constant *k*_F2_ could also be fitted but, in order not to bias the calculation of Φ values we chose not to calculate the free energy of unfolding in absence of denaturant, Δ*G*_D-N_, from the kinetic data. For calculations of Φ values we instead used ΔΔ*G*_D-N_ values obtained from the midpoints of the equilibrium experiments and assuming a similar *m*_D-N_ value in the curve fitting, for wild type and amide-to-ester mutants (1.0 kcal mol^−1^ M^−1^, from experiments with Y190W) (Table 1). Fitting of kinetic folding rate constants was done using Eq. 1. Calculated Φ-values were similar whether or not a shared *m*_D-N_ value was assumed.

**Table 1:** Equilibrium and kinetic parameters for wild type and amide-to-ester mutants of PSD-95 PDZ2.

PSD-95 PDZ2 variant	ΔΔ*G*_D-N_ 1 (kcal mol^−1^)	*k*_F1_ (s^−1^)	*k*_U2_ (s^−1^)	Φ_TS1_ 3	Φ_TS2_ 4
Y190W	−0.56±0.27	3.3±0.3	0.016±0.003	−0.16±0.17^5^	0.20±0.14^5^
V178C/Y190W^6^	0	3.9±0.13	0.041±0.006		
L170λ	0.93±0.31	3.8±0.2	0.14±0.01	0.01±0.07	0.15±0.06
G171γ	0.29±0.35^2^	5.5±0.4	0.20±0.02		
F172φ	1.43±0.67	4.6±0.4	1.20±0.09	−0.07±0.08	−0.64±0.31
I174ι	1.51±0.87	5.9±0.6	0.39±0.04	−0.17±0.12	0.0±0.1
G176γ	∼0^2^	2.5±0.2	0.062±0.014		

1 The *m*_D-N_ value used in the curve fitting (1.0 kcal mol^−1^ M^−1^) was determined from the Y190W data set, which displayed well-defined native and denatured baselines.

2 Too low to calculate a reliable Φ value.

3 Φ_TS1_ values were calculated in the absence of urea.

4 Φ_TS2_ values were calculated at [urea] = 6 M.

5 Reports on the side chain mutation V178C.

6 Pseudo wild type.

Truncation of side-chains in the core of a protein usually results in a decrease of the overall stability at equilibrium, Δ*G*_D-N_. In the case of a two-state folding process, Δ*G*_D-N_ is dependent on the forward and reverse rate constants for folding, *k*_F_ and *k*_U_. Usually, mutations of side-chains result in a decrease of *k*_F_ and/or an increase in *k*_U_ such that the resulting Φ values are between 0 and 1 (see methods). None of the amide-to-ester mutations in the present study led to a decrease in refolding rate constant, except possibly for G176γ (Fig. 3). In fact, the refolding rate constants increased for three of the mutants as compared to PSD-95 PDZ2 V178C/Y190W, suggesting that the hydrogen bonds, which are removed by the mutations, are involved in kinetically unfavourable interactions in the transition state for folding. Three backbone mutants, G171γ, F172φ and I174ι, displayed a significant increase in *k*_F_. However, while speeding up the folding reaction, the perturbation of these hydrogen bonds destabilizes the native state as reflected in the increased *k*_U_ values (Fig. 3, Table 1). Three mutants, L170λ, F172φ, I174ι, displayed ΔΔ*G*_D-N_ values that were sufficiently large (>0.6 kcal mol^−1^) [2] to calculate Φ values. Generally, Φ values were close to zero for the amide-to-ester mutations both for transition state 1 (TS1) and TS2 (Table 1). However, the Φ value for F172φ changes from only slightly negative in TS1 (−0.07±0.08) to −0.6±0.3 in TS2.

## Discussion

Mutations of side-chains have in combination with simulation shaped our knowledge about the protein folding reaction [1], [28]–[30]. On the other hand, there are only very few studies employing backbone mutations to study protein folding, following the first papers more than 10 years ago [4]–[6]. The primary reason is that amide-to-ester mutations and similar modifications of the backbone are still far from trivial to introduce in proteins. Yet they provide the possibility to probe the energetics of a fundamental aspect of protein structure, the hydrogen bond.

We have removed or destabilized hydrogen bonds from a β-sheet and from the connecting loop in PSD-95 PDZ2 by introduction of backbone amide-to-ester mutations (Fig. 1) using expressed protein ligation [21], [31]. The backbone hydrogen bonds in the β-sheet contribute significantly to the global stability of this protein domain (Table 1) and/or the local structure in case of the carbonyl group of Ala175 (see Results section). Moreover, the L170λ mutation in the loop resulted in global destabilization of close to one kcal mol^−1^ suggesting that at least one of the two putative hydrogen bonds depicted in Fig. 1C contributes to the stability of the folded state. The G171γ mutation also potentially targets two hydrogen bonds (see Results section and Fig. 1C). While the small value of ΔΔ*G*_D-N_ suggests a minor contribution to overall stability, the large error precludes a quantitative assessment. However, the more accurately determined increase in the *k*_U2_ value upon G171γ mutation suggests that the putative hydrogen bond(s) contribute to the stability of the domain. Polar groups are solvated in the denatured state. In order to achieve a stable fold, it is important to replace each desolvated hydrogen bond with an internal hydrogen bond in the folded state. Thus, if we count the hydrogen bonds (hydrogen bond inventory) we expect to have a similar or even identical number on each side of the folding reaction, or the reaction will become energetically unfavourable. The details of such energetics can be rather complex [32] and the actual contribution of a particular hydrogen bond to overall stability of a protein is therefore complex to deduce and highly context-dependent [3]. Nevertheless, our results for PSD-95 PDZ2 (observed ΔΔ*G*_D-N_ around 1 kcal mol^−1^) agree well with those of previous studies using backbone modifications.

In examining the folding kinetics we make the interesting observation that mutation of three of the backbone peptide bonds resulted in a slightly increased folding rate constant (Table 1, Fig. 3). Such kinetics result in Φ values <0 and has been observed for mutations involving truncations of side-chains (For example refs. [33], [34]) but also for a thioether backbone mutation in the YAP WW domain probing formation of a β-hairpin [5]. One likely interpretation of an increase in *k*_F1_ is that non-native interactions involving the targeted hydrogen bond donors and/or acceptors are formed in the initial transition state for the folding reaction of the wild type protein [35]. Removal of the non-native interaction speeds up folding. Such non-native interactions might thus reflect a frustrated energy landscape and could involve misaligned β-strands as suggested for a circularly permuted PDZ domain [18]. In particular, the large negative Φ value resulting from the F172φ mutation suggests a selective stabilization of TS2, which is not present in the ground states.

**Figure 4:**
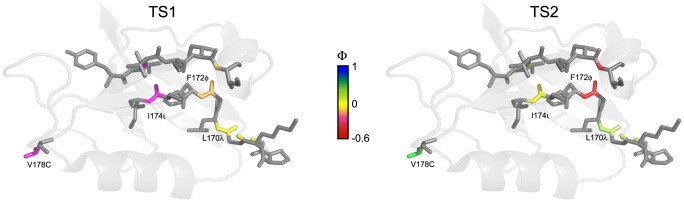
Native and non-native bond formation in the transition states for folding of PSD-95 PDZ2. Φ-values are generally very low but tend to increase from TS1 (left panel) to TS2 (right panel). The exception is the F172φ mutation, which is going from a slightly negative value to a highly negative Φ-value in TS2. The ΔΔ*G*_D-N_ values of G171γ and G176γ were too low to allow calculation of Φ-values.

The structures of two consecutive rate-limiting barriers were previously deduced based on Φ-value analyses of two different PDZ domains, PTP-BL PDZ2 [17] and PSD-95 PDZ3 [16]. These studies show that the late transition state (TS3 in Fig. 3D) has native-like side-chain interactions in large parts of the PDZ domain structure. Folding nuclei were identified in the strands β1, β4 and β6 for PTP-BL PDZ2 and in α2, β5 and β6 for PSD-95 PDZ3. We later identified a very early transition state (TS1) in the folding of PDZ domains [15] and redefined the numbering of transition states according to Fig. 3D. The compactness in terms of *β*_T_ value and the Φ value analyses of the later transition states (TS2 and TS3) all suggest that the structure of the first transition state TS1 is highly heterogeneous. The current study addresses formation of backbone hydrogen bonds in TS1 and TS2, in the strands β2 and β3, as well as the loop connecting β1 and β2 and the helix α1 (Fig. 4). We find very low Φ values for the backbone hydrogen bonds in this region for the two early transition states (TS1 and TS2), in agreement with the previous studies [16], [17]. Due to the relatively low thermodynamic stability of the amide-to-ester mutants of PSD-95 PDZ2, we could not measure Φ-values for the late, more native-like third transition state (TS3).

The amide-to-ester mutations are all in the ligand-binding groove. Mutations in this region have shaped the broad but overlapping ligand specificity in the PDZ family. It is reasonable that the ligand-binding groove of PDZ domains, which is optimized for function, should not affect the folding pathway, since this could lead to misfolding. Thus, while other parts of the PDZ domain govern early events in the folding reaction of PDZ domains [16]–[18], the ligand-binding groove may accept thermodynamically unfavourable amino acid side-chains, such as the conserved His in α2 [16], [36], allowing for evolution of new specificities [37], [38].
